# The Joint Effect of Perceived Psychosocial Stress and Phthalate Exposure on Hormonal Concentrations during the Early Stage of Pregnancy: A Cross-Sectional Study

**DOI:** 10.3390/children9101561

**Published:** 2022-10-15

**Authors:** Henrieta Hlisníková, Miroslava Nagyová, Branislav Kolena, Miloš Mlynček, Tomáš Trnovec, Ida Petrovičová

**Affiliations:** 1 Department of Zoology and Anthropology, Faculty of Natural Sciences and Informatics, Constantine the Philosopher University in Nitra, 94974 Nitra-Chrenová, Slovakia; 2 Department of Nursing, Faculty of Social Sciences and Health Care, Constantine the Philosopher University in Nitra, 94974 Nitra-Chrenová, Slovakia; 3 Department of Environmental Medicine, Faculty of Public Health, Slovak Medical University, 83101 Bratislava, Slovakia

**Keywords:** phthalate exposure, psychosocial stress, hormones, pregnant woman

## Abstract

Phthalates alter the hormonal balance in humans during pregnancy, potentially affecting embryonic and fetal development. We studied the joint effect of exposure to phthalates, quantified by urinary phthalate metabolite concentration, and perceived psychological stress on the concentration of hormones in pregnant women (*n* = 90) from the Nitra region, Slovakia, up to the 15th week of pregnancy. We used high-performance liquid chromatography, tandem mass spectrometry (HPLC-MS/MS), and electro-chemiluminescence immunoassay to determine urinary concentrations of phthalates and serum concentrations of hormones, respectively. We used Cohen perceived stress scale (PSS) to evaluate the human perception of stressful situations. Our results showed that mono(carboxy-methyl-heptyl) phthalate (cx-MiNP) and a molar sum of di-iso-nonyl phthalate metabolites (ΣDiNP) were negatively associated with luteinizing hormone (LH) (*p* ≤ 0.05). Mono(hydroxy-methyl-octyl) phthalate (OH-MiNP) and the molar sum of high-molecular-weight phthalate metabolites (ΣHMWP) were positively associated with estradiol (*p* ≤ 0.05). PSS score was not significantly associated with hormonal concentrations. When the interaction effects of PSS score and monoethyl phthalate (MEP), cx-MiNP, ΣDiNP, and ΣHMWP on LH were analyzed, the associations were positive (*p* ≤ 0.05). Our cross-sectional study highlights that joint psychosocial stress and xenobiotic-induced stress caused by phthalates are associated with modulated concentrations of reproductive hormones in pregnant women.

## 1. Introduction

Prenatal development is a complex process regulated by genetic and hormonal factors and the environment of the mother and fetus [1]. The prenatal period, especially the early stage of pregnancy, is dependent on the maternal endocrine system [2]. The maternal endocrine system changes rapidly during pregnancy [3] and can be affected by various environmental factors, such as exposure to environmental chemicals with further adverse effects on the developing fetus [4].

Phthalates are chemicals used in the plastic industry to soften plastic materials [5]. They are primarily used in polyvinyl chloride products [6]. People are exposed to phthalates ubiquitously. They act as endocrine disruptors in the human body affecting the physiological hormonal balance of the organism [7], such as decreased maternal concentrations of testosterone [8], thyroid hormones [9], fetal concentrations of cortisol [10] as well as increased maternal concentrations of estradiol [8]. Moreover, phthalates can pass the placental barrier and affect fetal development and health [11], resulting in adverse pregnancy outcomes [12], as well as in numerous reproductive [13] and neurodevelopmental disorders of progeny [14].

Psychosocial stress during pregnancy is one of the most significant environmental factors inducing an imbalance in the maternal endocrine system. Stress is regulated by various physiological processes trying to maintain the dynamic balance of the organism. The essential constituent of the stress response is the hypothalamic-pituitary-adrenal (HPA) axis [15], regulated by the hypothalamic paraventricular nucleus. Neurons in that region secret corticotropin-releasing hormone (CRH), stimulating the secretion of adrenocorticotropic hormone (ACTH) in the anterior lobe of the pituitary gland. ACTH induces cortisol secretion in the adrenal gland. Cortisol in the bloodstream inhibits the further secretion of CRH and ACTH from the hypothalamus and pituitary gland by negative feedback [16]. However, the stress response does not affect only the secretion of cortisol. The HPA axis can be modulated by the activity of the hypothalamic-pituitary-thyroid (HPT) and hypothalamic-pituitary-gonadal (HPG) axes. CRH interacts with hypothalamic neurons secreting gonadotropin-releasing hormone (GnRH) and thyrotropin-releasing hormone (TRH), resulting in the inhibition of luteinizing hormone (LH) and thyroid-stimulating hormone (TSH) secretion of the pituitary gland. This interaction is linked with decreased sex steroids and thyroid hormones [17,18]. Previous studies have observed the associations between perceived stress and modulated hormonal concentrations [19], resulting in preterm birth and low birth weight [20], as well as impairment of reproductive and neural health of progeny [21].

Although perceived psychosocial stress and exposure to chemicals are associated with similar adverse health outcomes, only a few studies have focused on the joint effect of xenobiotic-induced stress and psychosocial stress on women’s health during pregnancy. We considered an investigation of such a combination of stressors as indicated due to the possible significant magnification of adverse health effects. Our study aimed to determine the association of joined effect of phthalate metabolites with perceived psychological stress (PSS) score on hormonal concentrations.

## 2. Materials and Methods

### 2.1. Study Population

The present cross-sectional study is a part of the Mother–Infant Study Cohort (PRENATAL), designed to investigate the association between maternal phthalate exposure and reproductive and neurobehavioral outcomes of progeny. The study population consisted of pregnant women up to the 15th week of pregnancy (*n* = 90) from the Nitra region, Slovakia. The research was conducted with the approval of the University Hospital Ethics Committee in Nitra. Participation was anonymous and voluntary, and all probands signed informed consent prior to involvement. The sample collection and exclusion criteria were described elsewhere [22].

### 2.2. The Questionnaire Method of Data Collection

A trained technician completed the questionnaires to obtain essential data on health conditions, previous pregnancies, and baseline characteristics during the early stage of the pregnancy visit. We used the Cohen perceived stress scale-10 (PSS-10) to evaluate the human perception of stressful situations. This scale contains ten questions, four are formulated positive, and six are negative. For each question, the proband chooses one of five possible answers: never, rarely, occasionally, quite often, and often. Each question is scored on a 5-point scale that ranges from never (0) to frequently (4). Positively formulated items are reversed. The final score for an average person without chronic stress or stress-related illness is around 13 points. A stress-exposed person scores an average of 20 points or more [23]. Based on this score, the cohort of pregnant women was divided into two groups- low (≤19 points) and high stressed probands (≥20 points).

### 2.3. Qualitative and Quantitative Analysis of Phthalate Metabolites from Urine Spots

The qualitative and quantitative analysis of phthalate metabolites has been described elsewhere [24]. Briefly, we used high-performance liquid chromatography (HPLC) and tandem mass spectrometry (MS/MS) (Infinity 1260 and 6410 triplequad, Agilent, Santa Clara, CA, USA) to quantify the urinary concentration of 17 phthalate metabolites by the method built on the basis of previously published offline SPE and online HPLC-MS/MS methods [25,26]. The analysis was performed in Physiological Analytical Laboratory, Constantine the Philosopher University in Nitra. Our laboratory passed interlaboratory tests in the HBM4EU QA/QC program (HBM4EU). Internal quality control was performed by analyses of 2 control materials (a mixture of urine samples) with known concentrations (lower and higher concentrations). The limits of quantification (LOQ) were estimated based on the lowest quantifiable concentration of the standard in the calibration curve individually for each phthalate metabolite. LOQs were estimated between 1 and 2.5 ng/mL. Precursor and product ions and LOQs are shown elsewhere [24].

### 2.4. Qualitative and Quantitative Analysis of Maternal Hormonal Concentrations from Blood Serum Spots

Quantitative determination of follicle-stimulating hormone (FSH), luteinizing hormone (LH), estradiol, testosterone, cortisol, thyroid-stimulating hormone (TSH), free thyroxine (FT4), and free triiodothyronine (FT3), was performed automatically by electro-chemiluminescence immunoassay (Elecsyssystem; Roche, Basel, Switzerland) in immunoassay system from human serum [27].

### 2.5. Statistics

For the values below the LOQ of phthalate metabolite concentrations, we imputed by taking the LOQ value divided by the square root of 2 if concentrations had <20% of samples below the LOQ and the LOQ divided by 2 if >20% of samples fell below the LOQ. Only those phthalate metabolites whose concentrations were at least in 70% of samples above the LOQ were included in the statistical analyses.

We calculated the molar sum of di-iso-butyl phthalate metabolites (ΣDiBP = MiBP + OH-MiBP), di-*n*-butyl phthalate metabolites (ΣDnBP = MnBP + OH-MnBP), di(2-ethylhexyl) phthalate metabolites (ΣDEHP = OH-MEHP + oxo-MEHP + cx-MEPP), di-iso-nonyl phthalate metabolites (ΣDiNP = OH-MiNP+ cx-MiNP), low molecular-weight phthalate metabolites (ΣLMWP = MEP + MiBP + MnBP + OH-MiBP + OH-MnBP), and high molecular-weight phthalate metabolites (ΣHMWP = OH-MEHP + oxo-MEHP + cx-MEPP + OH-MiNP + cx-MiNP). The concentrations of phthalate metabolites, their molar sums, and hormones were log-transformed for the statistical analysis because of the non-normal data distribution.

**Figure 1:**
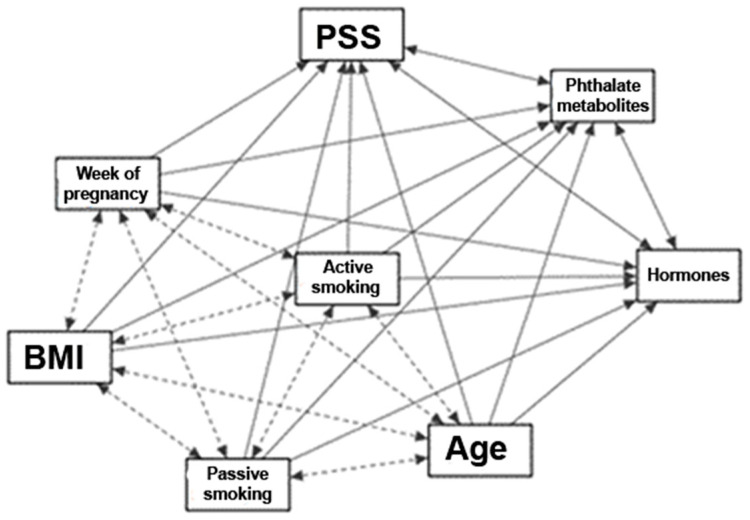
Path diagram explaining the potential associations between the concentrations of hormones, phthalate metabolites, PSS score and confounding variables (age, BMI, week of pregnancy, active and passive smoking). Solid arrow represents association with between the main variables (concentrations of hormones, phthalate metabolites, PSS score) or between confounding variable and main variables. Interrupted arrow represents association between confounding variables.

Pearson’s correlation analysis, unpaired *t*-test, and one-way analysis of variance (ANOVA) were used to determine confounding variables from probands’ baseline characteristics. We analyzed the following numeric variables: week of pregnancy at the time of sample collection, age, BMI, number of previous pregnancies, and nominal variables: sex of the child, active and passive smoking, education, and living area. The following significant (*p* ≤ 0.05) confounding variables were detected: week of pregnancy at the time of sample collection, age, BMI, and active and passive smoking. We created a Path diagram (Figure 1) to visualize the potential associations between exposure (phthalate metabolites, perceived stress), outcome (hormonal concentrations), and confounding variables (age, week of pregnancy, BMI, active and passive smoking).

**Figure 2:**
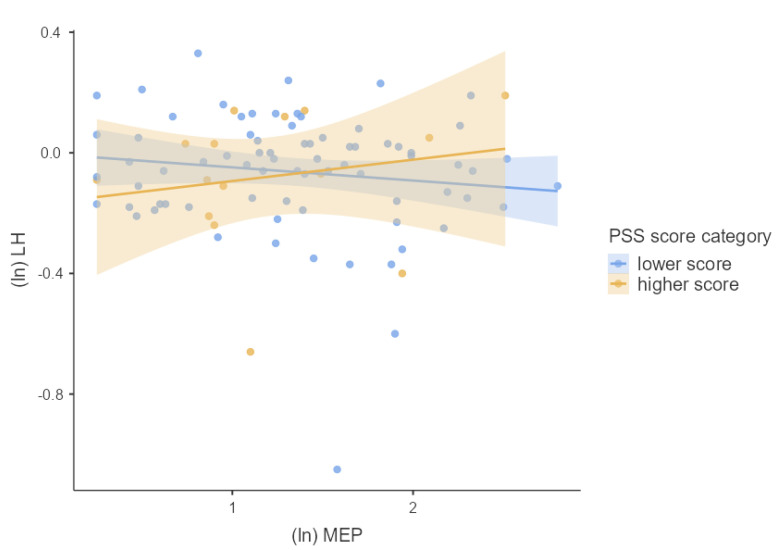
The associations between the concentrations of LH and MEP based on the PSS score category stratification; Legend: ln—log transformation, LH—luteinizing hormone, MEP—monoethyl phthalate, PSS—perceived stress scale.

**Figure 3:**
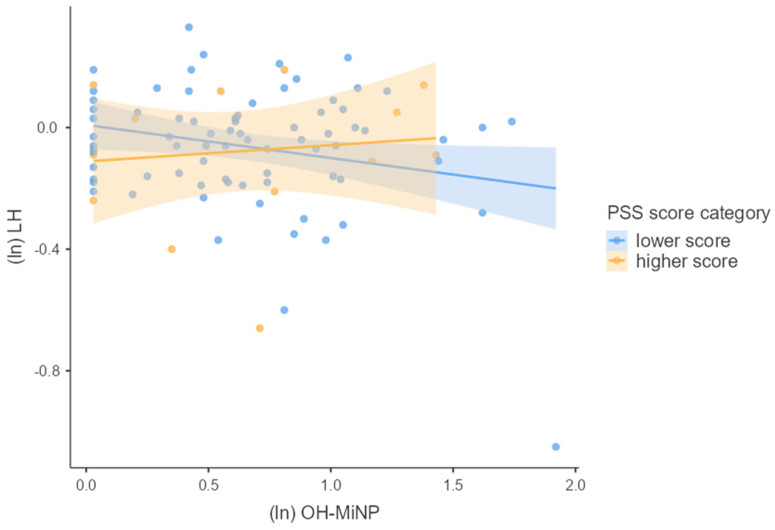
The associations between the concentrations of LH and OH−MiNP based on the PSS score category stratification; Legend: ln—log transformation, LH—luteinizing hormone, OH−MiNP—mono(hydroxyl−methyl−octyl) phthalate, PSS—perceived stress scale.

**Figure 4:**
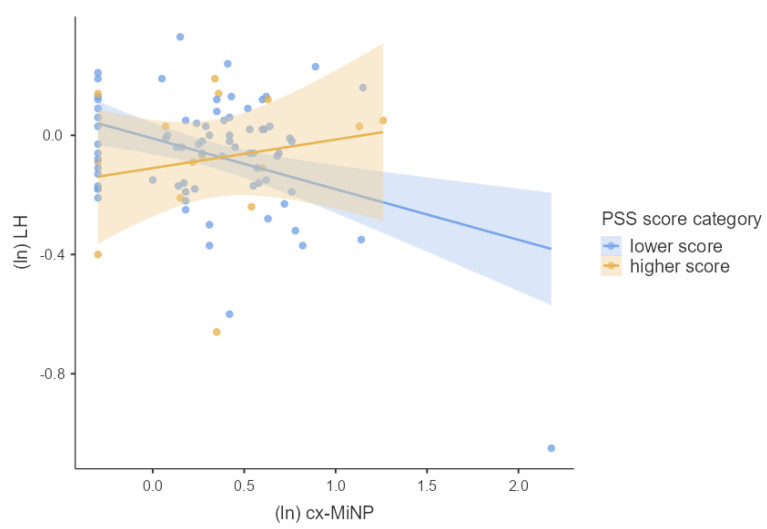
The associations between the concentrations of LH and cx−MiNP based on the PSS score category stratification; Legend: cx-MiNP—mono(carboxy−methyl−heptyl) phthalate, ln—log transformation, LH—luteinizing hormone, PSS—perceived stress scale.

**Figure 5:**
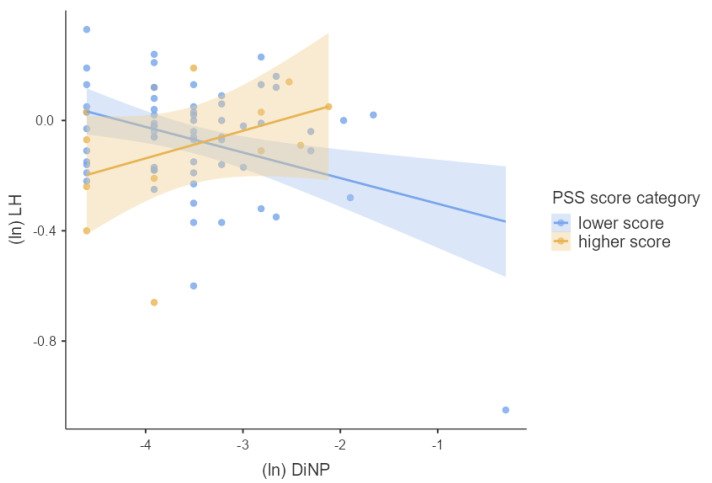
The associations between the concentrations of LH and DiNP based on the PSS score category stratification; Legend: DiNP—molar sum of di−iso−nonyl phthalate metabolites, ln—log transformation, LH—luteinizing hormone, PSS—perceived stress scale.

**Figure 6:**
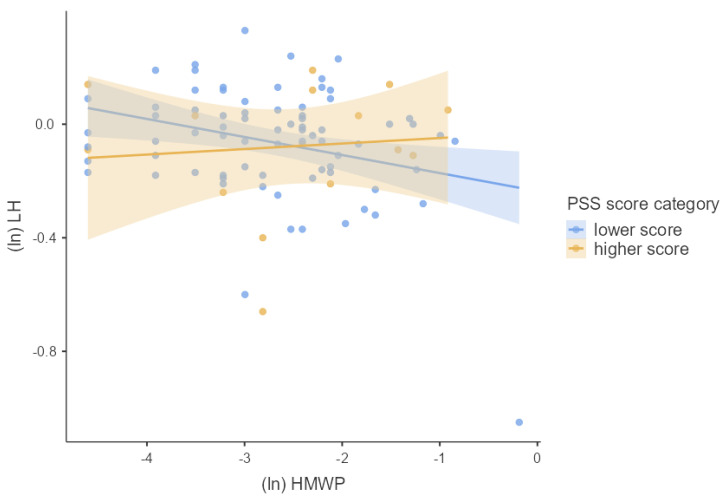
The associations between the concentrations of LH and HMWP based on the PSS score category stratification; Legend: HMWP—molar sum of high−molecular−weight phthalate metabolites, ln—log transformation, LH—luteinizing hormone, PSS—perceived stress scale.

We first tested the main effects of phthalate metabolite concentrations and PSS score separately using multiple linear regression adjusted for confounders. Next, we used multiple linear regression to test whether phthalate metabolite concentrations interacted with the PSS score to predict the hormonal concentrations of pregnant women. For this purpose, we used guidelines for interaction effects provided by Aiken and West [28] described in Schreier et al. [29]. To visualize our results, we used general mixed models. We divided our cohort into two groups based on the height of the PSS score (lower and higher PSS score). The associations between hormonal concentrations and concentrations of phthalate metabolites using general mixed models for each group were plotted in Figure 2, Figure 3, Figure 4, Figure 5 and Figure 6. We used IBM SPSS Statistics (version 21.0; SPSS Inc., Chicago, IL, USA) and jamovi for statistical analysis. The effect size was considered statistically significant when the *p* ≤ 0.05.

## 3. Results

### 3.1. Demographic Characteristics

The cohort (PRENATAL) consisted of 90 women up to the 15th week of pregnancy from the Nitra region, Slovakia. Their average age reached 30.80 ± 4.97 years, and the average week of gestation was 10.46 ± 1.80 weeks. Their average PSS score was 15.20 ± 4.82 points which is considered normal. The descriptive characteristics of our cohort are shown in Table 1.

### 3.2. Biomonitoring of Phthalate Metabolites

We found that the concentrations of detected phthalate metabolites were above the LOQ in the following descending order: cx-MEPP (96.59%) > MEP > OH-MnBP > MiBP > MnBP > OH-MEHP > oxo-MEHP > OH-MiNP > cx-MiNP > OH-MiBP > MBzP > MMP > oxo-MiNP > MnPeP, MCHP, MiNP, MnOP (0.00%). The highest median value was reached by the metabolite MEP (20.90 ng/mL), and the lowest median concentration above the LOQ was reached by the metabolite MBzP (1.17 ng/mL). The descriptive statistics of phthalate metabolites are shown in Table 2.

### 3.3. Analyses of Hormones

Table 3 shows descriptive statistics on serum hormones. We compared observed concentrations with reference values for pregnant women (www.perinatology.com, 12 March 2021). The serum concentrations of hormones detected were above the reference values in the following descending order: estradiol (64.77%) > cortisol > LH > TSH > FT4 > FT3 > FSH, testosterone (0.00%). The serum concentrations of hormones detected were below the reference values in the following descending order: FT3 (94.32%) > FT4 > TSH > cortisol > estradiol, FSH, LH, testosterone (0.00%).

### 3.4. Associations between Phthalates, Hormones, and Perceived Stress

We analyzed the relationships between log-transformed concentrations of phthalate metabolites and log-transformed hormonal concentrations using multiple linear regression adjusted for confounding variables (week of pregnancy at the time of sample collection, age, BMI, and active and passive smoking) (Table S1 in Supplementary Data). We noticed significant positive associations between OH-MiNP (β = 0.237, *p* = 0.015), ∑HMWP (β = 0.233, *p* = 0.019) and estradiol; and significant negative association between cx-MiNP (β = −0.225, *p* = 0.037) and LH. We reported negative association on the border of significance between ∑DiNP (β = −0.228, *p* = 0.054) and LH.

To examine the perception of psychosocial stress, we used a questionnaire examination method, namely the Cohen perceived stress scale (PSS), which consists of 10 questions. The final test score is the sum of points for all questions in the test. The higher the score, the greater the chance that the proband experiences a higher level of stress, which could be associated with a disturbance of hormonal balance. We analyzed the relationships between PSS score and hormonal concentrations using multiple linear regression adjusted for confounding variables (Table S1 in Supplementary Data). We did not observe any significant association between PSS score and hormonal concentrations in adjusted models.

We investigated whether log-transformed concentrations of phthalate metabolites and psychosocial stress interacted to affect the log-transformed hormonal concentrations in pregnant women by multiple linear regression adjusted for confounding variables (week of pregnancy at the time of sample collection, age, BMI, and active and passive smoking). There was significant PSS score × MEP (β = 0.218, *p* = 0.042), OH-MiNP (β = 0.255, *p* = 0.016), oxo-MiNP (β = 0.350, *p* = 0.001), cx-MiNP (β = 0.349, *p* = 0.001), ΣDiNP (β = 0.329, *p* = 0.002), ΣHMWP (β = 0.226, *p* = 0.039) interaction effects on LH. The results of multiple linear regression are shown in Table S1 in Supplementary Data. As can be seen in Figure 2, Figure 3, Figure 4, Figure 5 and Figure 6, there is an antagonistic effect of phthalate metabolites (MEP, OH-MiNP, cx-MiNP, ΣDiNP, ΣHMWP) on concentrations of LH based on the height of PSS score. In the group of probands with higher PSS score, there is a positive association between levels of phthalate metabolites and LH, while a negative association can be observed in the less stressed group of probands.

When comparing the difference between the main effect of PSS score or phthalate metabolites separately and their interaction effect on hormonal concentrations, the associations with estradiol disappeared. Contrary, more associations with LH appeared significant, but they changed their direction from negative to positive associations.

## 4. Discussion

Several studies have simultaneously examined xenobiotic-induced and psychosocial stress in pregnant women [29,30,31,32]. To our knowledge, only one examined the effects of such stressors on the modulation of hormone concentration in pregnant women [29]. Schreier et al. [29] noticed that higher mercury concentrations could result in decreased cortisol concentrations in the morning but only in stress-exposed pregnant women from Mexico City (*n* = 732). Our study probably is the first to examine the joint effect of phthalate metabolites and psychosocial stress on hormonal concentrations during pregnancy. It focuses on such relationships in view of the association between the health during pregnancy and postnatal health of the progeny with the maternal hormonal system.

A strong relationship between phthalate exposure and disruption of hormonal concentrations in pregnant women has been previously reported [8,33]. In addition to these observations, our data suggest that these relationships may be modified by perceived stress. Our study revealed positive and negative associations between the concentrations of phthalate metabolites and estradiol, LH, respectively. We also observed associations between the PSS score x phthalate metabolite interactions and concentration of LH.

### 4.1. Associations between Phthalate Exposure and Hormonal Concentrations

We observed a negative association between LH and cx-MiNP, ∑DiNP. Al-Saleh [34] showed non-significant positive associations between phthalate metabolites and levels of LH in Saudi women (*n* = 523) undergoing in vitro fertilization. Higher levels of oxo-MEHP were associated with higher LH in women (*n* = 58) and men (*n* = 48) aged 11–88 years from China during summer but not during winter [35]. Contrary, the study of Wen et al. [36] noticed the inverse association between DEHP metabolites and LH in pubertal boys and girls (*n* = 239) in Taiwan; however, this association was significant only in boys.

Our results have shown that OH-MiNP, ΣDiNP, and ΣHMWP were positively associated with estradiol levels. However, the results of other studies are inconsistent. According to Sathyanarayana et al. [8], MiBP, MBzP, MEHP, and oxo-MEHP were associated with increased estradiol levels during early pregnancy in pregnant women from the TIDES cohort (*n* = 591). On the contrary, Cao et al. [37] noticed an inverse association between LMWP metabolites and estradiol in women (*n* = 246) from China. Interestingly, the study of Johns et al. [33] reported non-significant positive and negative associations between estradiol and levels of LMWP and HMWP metabolites, respectively, in pregnant women from Puerto Rico (*n* = 106).

The inconsistencies in associations between phthalate metabolites and reproductive hormone concentrations (LH, estradiol) may be attributed to the various population groups in these studies. There is a difference in reproductive physiology in men and women, as well as in humans during puberty and adulthood [38]. This may lead to different associations between phthalate metabolites and reproductive hormones. The next reason for the inconsistent results may be the different estrogenic activity based on the group of phthalate diesters and their metabolites. The basic chemical structure of most phthalate metabolites is the same. It consists of the benzene ring. However, the metabolites differ in the side chain length, which could lead to different physicochemical properties and different mechanisms of toxicity in the body [39,40]. Phthalate exerting estrogenic activity, such as DiNP metabolites in our study, could stimulate the estrogen receptor or estradiol synthesis, leading to decreased LH via a negative feedback loop within the HPG axis. On the contrary, phthalate exerting anti-estrogenic activity could block the estrogen receptor or inhibit estradiol synthesis, leading to increased LH [41,42,43].

### 4.2. Joined Effect of Phthalate Exposure and Perceived Psychosocial Stress on Hormonal Concentrations

Although we did not find any significant associations between PSS score and hormones, we observed a significant positive association with LH when we evaluated the interaction between PSS score and phthalate metabolites. Surprisingly, when we evaluated the association between DiNP metabolites and LH separately without a PSS score, we noticed a negative association. In contrast, when we assessed the interaction PSS score × phthalate metabolites, the direction of the association with LH changed to a positive association. When we divided probands based on the PSS score into two groups (lower and higher PSS score), we observed an antagonistic effect of phthalate metabolites based on the height of the PSS score. In the group of probands with higher PSS score, there was a positive association between levels of phthalate metabolites and LH. In comparison, a negative association was observed in probands with a lower score.

Published studies on the effect of perceived psychosocial stress on the endocrine system showed inconsistent results. High stress levels during pregnancy were associated with increased serum cortisol and CRH concentrations [44]. However, Braig et al. [45] did not observe significant correlations between self-reported psychosocial stress and hair cortisol in women. Interestingly, Pruessner et al. [46] showed that chronic stress was associated with decreased cortisol concentration. Perceived stress, particularly chronic stress, can both decrease and increase cortisol concentration [46]. There are several reasons why chronic stress could be associated with elevated and decreased cortisol levels, such as cortisol depletion, lack of free cortisol, impaired cortisol secretion regulating hormones (ACTH, CRH), or modulated glucocorticoid receptor sensitivity [47]. The stress response involves not only the HPA axis but also HPG and HPT axes. CRH from the HPA axis inhibits HPG and HPT axes [18]. Chronic stress and higher cortisol levels are associated with fertility disorders in females, both in humans and animals, such as premature ovarian failure, which is linked with increased concentrations of FSH and decreased concentrations of LH, estradiol and testosterone [48,49,50]. We observed the opposite trend in probands with higher PSS score, who had lower cortisol and higher LH concentrations, compared to probands with lower PSS score. The study of Breen and Mellon [51] pointed to the inverse relationship between cortisol and LH. Higher cortisol levels directly inhibit pituitary gonadotropin levels, so we hypothesize that LH could not be inhibited in probands with higher PSS score due to lower cortisol concentrations compared to probands with lower PSS score. Our hypothesis could be confirmed by a study showing that high levels of gonadotropins were observed in subjects diagnosed with decreased cortisol levels without hormonal replacement therapy [52].

We assume that xenobiotic-induced stress represented by phthalate exposure and psychosocial stress share a similar target which is hormonal balance. Several plausible mechanisms of action of phthalates and psychosocial stress can be suggested. One of them is the modulation of the synthesis and metabolism of hormones, leading to changes in HPA, HPG, and HPT feedback loops [15,53]. We have shown that the joint effect of psychosocial stress and phthalate metabolites is associated with the modulation of LH. Interestingly, we observed a more significant effect of phthalates and PSS score in the interaction models compared to their separate main effects on LH concentrations. Several systematic and literature reviews have followed a similar pattern. Psychosocial and xenobiotic stress cause a more significant effect on health outcomes (e.g., birth weight, neurological parameters, obesity, respiratory diseases) compared to their individual effects [54,55,56,57]. A possible explanation for this synergism is that psychosocial stress increases the sensitivity of the organism to xenobiotics [58].

Although no study has examined the relationship between phthalate exposure, psychosocial stress, and hormonal concentrations during pregnancy, some studies lacking hormonal data have observed the effects of prenatal phthalate exposure and maternal stress on pregnancy outcomes and neonatal health. According to Ferguson et al. [32], exposure to stressful life events (SLEs) increased the significance of the association between exposure to DEHP during the third trimester of pregnancy and preterm birth (*n* = 783) in the TIDES cohort. Moreover, the TIDES cohort reported that exposure to SLEs during the first trimester of pregnancy (*n* = 738) was associated with non-significant positive relations between phthalate exposure and reproductive biomarkers (e.g., anogenital distance, anoscrotal distance, anopenile distance) in male newborns. On the contrary, in the group of pregnant women with no exposure to SLEs was observed significant negative associations between reproductive biomarkers and phthalate exposure in male newborns [31]. The opposite pattern was observed in the MIREC cohort [30], wherein the lower stressor group was noted the positive association between phthalate metabolites with androgen-disrupting activity and anopenile distance in male newborns (*n* = 147). Interestingly, in the MIREC cohort, there was a significant positive association between phthalate metabolites with androgen-disrupting activity and reproductive biomarkers in female newborns (*n* = 153) but only in the higher stressor group [30]. Pregnancy and newborn outcomes, such as birth timing or reproductive biomarkers, are also associated with prenatal hormonal concentrations exposure [31]. The maternal and fetal endocrine system strictly regulates prenatal development. Therefore, any modulation in hormonal concentrations during pregnancy can potentially lead to other adverse outcomes [1].

The current study has a cross-sectional design in which exposure and outcome are assessed simultaneously and only allows hypotheses to be formulated but cannot define causal relationships. Subsequent case–control or prospective cohort studies will be needed to validate our hypotheses and the results. The next limitation of our study is the size of the cohort. Therefore, verifying our findings on a larger cohort of pregnant women is necessary. On the other side, the main conclusions having crucial public health significance are supported by convincing statistics and methods for stress assessment. The strength of using Cohen’s perceived stress test in our study is an interview approach by one training technician, which explained the items and questions that the subject might have otherwise misunderstood. Future research would benefit from including additional measures, such as physiological assessments, when assessing perceived stress. Additionally, using self-reported data introduces several limitations, such as response bias. Collecting only one urine sample to determine the concentration of phthalate metabolites during early pregnancy can also be considered a limitation of our study. However, some studies report that the concentrations of phthalate metabolites in repeated urine samples from a single proband were approximately in the same range [59]. It has also been confirmed that there was no significant difference in urinary phthalate metabolite concentrations in spot, morning void, 24 h or 48 h pooled urine samples [60,61].

## 5. Conclusions

We monitored the hormonal concentrations of pregnant women during the early stage of pregnancy in association with phthalate metabolites and perceived stress. Our results showed that OH-MiNP and ΣHMWP were positively associated with estradiol. Cx-MiNP and ΣDiNP were negatively associated with LH. PSS score was not significantly associated with hormonal concentrations. When the interaction effects of PSS score and MEP, cx-MiNP, ΣDiNP, and ΣHMWP on LH were analyzed, the associations were positive. We are the first to show that the joint effect of psychosocial stress and phthalate exposure in pregnant women is associated with a more significant modulation of the hormonal levels compared to the separate effects of phthalate metabolites and stress. During pregnancy, maternal hormonal balance is important for proper prenatal development [3]. Therefore, any modulation of hormonal balance (increase but also decrease in hormone concentration) due to exogenous factors can induce changes in maternal health and the health of future offspring [1]. Understanding the mechanisms by which the interaction between prenatal psychosocial stress and xenobiotic-induced stress may affect the endocrine system needs further study.

## Figures and Tables

**Table 1:** Characteristics of the cohort based on demography and perceived stress (*n* = 90).

		Mean (±SD)	% (*n*)
Age		30.80 ± 4.97	
BMI (n_miss_ = 2)		23.05 ± 4.07	
Week of pregnancy		10.46 ± 1.80	
PSS score		15.20 ± 4.82	
PSS score category	Lower score (≤19 points)		83.30% (*n* = 75)
	Higher score (≥20 points)		16.70% (*n* = 15)
Education (n_miss_ = 1)	High school		38.20% (*n* = 34)
	College/university		61.80% (*n* = 55)
Living area	Rural		54.40% (*n* = 48)
	Urban		45.60% (*n* = 40)
Number of previous pregnancies	0		50.00% (*n* = 45)
	1		28.90% (*n* = 26)
	2		14.40% (*n* = 13)
	≥3		6.70% (*n* = 6)
Active smoking	Smoker		5.60% (*n* = 5)
	Former smoker		28.90% (*n* = 26)
	Non-smoker		65.60% (*n* = 59)
Passive smoking (n_miss_ = 1)	Yes		20.22% (*n* = 18)
	No		79.78% (*n* = 71)

Legend: *n*—number of probands, n_miss_—number of missing values, PSS—the Cohen perceived stress scale, SD—standard deviation.

**Table 2:** Descriptive statistics of phthalate metabolite concentrations (ng/mL).

Compound Name	% ≥LOQ	MIN	MED	MAX	MEAN	SD
MMP	30.68	≤LOQ	≤LOQ	26.21	3.01	4.09
MEP	94.32	≤LOQ	20.90	629.28	55.78	95.12
MiBP	92.05	≤LOQ	14.04	420.07	24.85	47.88
MnBP	90.91	≤LOQ	17.17	415.45	27.40	48.09
OH-MiBP	77.27	≤LOQ	1.89	38.61	3.06	4.61
OH-MnBP	94.32	≤LOQ	6.94	132.97	10.34	16.19
MnPeP	0.00	≤LOQ	≤LOQ	≤LOQ	≤LOQ	0.00
MCHP	0.00	≤LOQ	≤LOQ	≤LOQ	≤LOQ	0.00
MBzP	52.27	≤LOQ	1.17	28.72	1.84	3.43
OH-MEHP	86.36	≤LOQ	4.02	43.20	6.09	6.74
oxo-MEHP	85.23	≤LOQ	3.30	25.73	4.95	4.99
cx-MEPP	96.59	≤LOQ	5.74	53.07	8.60	8.69
MiNP	0.00	≤LOQ	≤LOQ	≤LOQ	≤LOQ	0.00
OH-MiNP	82.95	≤LOQ	4.16	82.25	8.32	12.39
oxo-MiNP	29.55	≤LOQ	≤LOQ	63.41	1.96	6.70
cx-MiNP	78.41	≤LOQ	2.03	151.04	4.60	16.10
MnOP	0.00	≤LOQ	≤LOQ	≤LOQ	≤LOQ	0.00
ΣDiBP	-	0.01	0.09	2.45	0.16	0.28
ΣDnBP	-	0.01	0.11	2.03	0.17	0.25
ΣDEHP	-	0.01	0.05	0.41	0.07	0.07
ΣDiNP	-	0.01	0.03	0.94	0.05	0.10
ΣLMWP	-	0.04	0.41	3.40	0.63	0.68
ΣHMWP	-	0.01	0.08	1.04	0.11	0.13

Legend: ≤LOQ—values below the limit of quantification, cx-MEPP—mono(2-ethyl-5-carboxypentyl) phthalate, cx-MiNP—mono(carboxy-methyl-heptyl) phthalate, MAX—maximum, MBzP—monobenzyl phthalate, MCHP—monocyclohexyl phthalate, MED—median, MEP—monoethyl phthalate, MiBP—mono-iso-butyl phthalate, MMP—monomethyl phthalate, MIN—minimum, MiNP—mono-iso-nonyl phthalate, MnBP—mono-*n*-butyl phthalate, MnOP—mono-*n*-octyl phthalate, MnPeP—mono-*n*-pentyl phthalate, OH-MEHP—mono(2-ethyl-5-hydroxyhexyl) phthalate, OH-MiBP—mono(hydroxy-iso-butyl) phthalate, OH-MnBP—mono(hydroxy-*n*-butyl) phthalate, OH-MiNP—mono(hydroxyl-methyl-octyl) phthalate, oxo-MEHP—mono(2-ethyl-5-oxohexyl) phthalate, oxo-MiNP—mono(oxo-methyl-octyl) phthalate, SD—standard deviation, ΣDiBP—molar sum of di-iso-butyl phthalate metabolites (MiBP + OH-MiBP), ΣDnBP—molar sum of di-*n*-butyl phthalate metabolites (MnBP + OH-MnBP), ΣDEHP—molar sum of di(2-ethylhexyl) phthalate metabolites (OH-MEHP + oxo-MEHP + cx-MEPP), ΣDiNP—molar sum of di-iso-nonyl phthalate metabolites (OH-MiNP + oxo-MiNP + cx-MiNP), ΣLMWP—molar sum of low-molecular-weight phthalate metabolites (MMP + MEP + MiBP + MnBP + OH-MiBP + OH-MnBP + MBzP), ΣHMWP—molar sum of high-molecular-weight phthalate metabolites (OH-MEHP + oxo-MEHP + cx-MEPP + OH-MiNP + oxo-MiNP + cx-MiNP).

**Table 3:** Descriptive statistics of hormonal concentrations.

Compound Name	Units	MIN	MED	MAX	MEAN	SD
FSH	mIU/mL	0.00	0.01	0.37	0.04	0.06
LH	mIU/mL	0.09	0.93	2.12	0.94	0.36
Estradiol	pg/mL	851.00	3079.00	20,800.00	4007.12	3280.26
Testosterone	ng/mL	0.16	0.68	1.51	0.74	0.29
TSH	μIU/mL	0.03	1.44	3.90	1.66	0.94
FT3	pg/mL	0.99	3.30	10.40	5.25	1.03
FT4	ng/mL	0.08	0.82	3.82	1.68	0.54
Cortisol	μg/mL	0.85	17.53	198.00	84.99	25.26

Legend: FSH—follicle-stimulating hormone, FT3—free triiodothyronine, FT4—free thyroxine, LH—luteinizing hormone, MAX—maximum, MED—median, MIN—minimum, SD—standard deviation, TSH—thyroid-stimulating hormone.

## Data Availability

Not applicable.

## Supplementary Materials

The following supporting information can be downloaded at: https://www.mdpi.com/article/10.3390/children9101561/s1, Table S1: Regression analyses of main and interaction effects between phthalate metabolites, psychosocial stress, and hormones.
