# Supplementary material for: The Joint Effect of Perceived Psychosocial Stress and Phthalate Exposure on Hormonal Concentrations during the Early Stage of Pregnancy: A Cross-Sectional Study
Source: Children (Basel). 2022 Oct 15;9(10):1561. doi: 10.3390/children9101561 (PMC9601203; doi:10.3390/children9101561)
Supplement: Supplementary file 1 [file children-09-01561-s001.zip › children-1966576-supplementary.pdf]

Table S1. Regression analyses of main and interaction effects between phthalate metabolites, psychosocial stress, and hormones

		(ln) FSH		(ln) LH		(ln) estradiol		(ln) testosterone		(ln) TSH		(ln) FT3		(ln) FT4		(ln) cortisol	
		β (95% CI)	*p*	β (95% CI)	*p*	β (95% CI)	*p*	β (95% CI)	*p*	β (95% CI)	*p*	β (95% CI)	*p*	β (95% CI)	*p*	β (95% CI)	*p*
1	PSS	-0.049 (-0.039,0.024)	0.639	0.046 (-0.007,0.011)	0.674	0.029 (-0.010,0.013)	0.774	-0.049 (-0.010,0.007)	0.664	-0.024 (-0.020,0.016)	0.833	0.089 (-0.006,0.015)	0.406	0.081 (-0.008,0.018)	0.460	0.126 (-0.010,0.040)	0.245
	(ln) MEP	-0.061 (-0.349,0.200)	0.589	-0.080 (-0.105,0.051)	0.493	0.023 (-0.090,0.111)	0.832	0.080 (-0.048,0.096)	0.505	0.046 (-0.124,0.183)	0.705	-0.022 (-0.097,0.080)	0.851	0.041 (-0.095,0.135)	0.724	-0.063 (-0.280,0.161)	0.593
	PSS	-0.054 (-0.040,0.024)	0.613	0.035 (-0.007,0.010)	0.744	0.031 (-0.010,0.014)	0.761	-0.045 (-0.010,0.007)	0.692	-0.019 (-0.020,0.017)	0.869	0.090 (-0.006,0.015)	0.406	0.083 (-0.008,0.019)	0.454	0.128 (-0.010,0.041)	0.243
2	(ln) MEP	-0.068 (-0.361,0.194)	0.552	-0.096 (-0.110,0.045)	0.406	0.027 (-0.089,0.114)	0.806	0.082 (-0.048,0.098)	0.502	0.053 (-0.122,0.190)	0.666	-0.015 (-0.096,0.084)	0.895	0.046 (-0.094,0.139)	0.699	-0.052 (-0.270,0.172)	0.659
	PSS x (ln) MEP	0.067 (-0.042,0.082)	0.523	0.218* (0.001,0.035)	**0.042**	-0.037 (-0.027,0.019)	0.714	-0.041 (-0.019,0.013)	0.717	-0.098 (-0.050,0.019)	0.384	-0.042 (-0.024,0.016)	0.697	-0.023 (-0.029,0.023)	0.835	-0.083 (-0.069,0.030)	0.445
1	PSS	-0.049 (-0.039,0.024)	0.639	0.046 (-0.007,0.011)	0.674	0.029 (-0.010,0.013)	0.774	-0.049 (-0.010,0.007)	0.664	-0.024 (-0.020,0.016)	0.833	0.089 (-0.006,0.015)	0.406	0.081 (-0.008,0.018)	0.460	0.126 (-0.010,0.040)	0.245
	(ln) MnBP	0.084 (-0.202,0.460)	0.439	-0.156 (-0.160,0.027)	0.162	0.116 (-0.052,0.189)	0.260	0.058 (-0.065,0.109)	0.614	0.016 (-0.173,0.199)	0.889	-0.043 (-0.128,0.086)	0.701	-0.006 (-0.142,0.135)	0.960	-0.067 (-0.346,0.186)	0.551
	PSS	-0.055 (-0.041,0.025)	0.613	0.028 (-0.008,0.010)	0.797	0.014 (-0.011,0.013)	0.895	-0.037 (-0.010,0.007)	0.750	0.005 (-0.018,0.019)	0.963	0.105 (-0.006,0.016)	0.342	0.099 (-0.008,0.020)	0.378	0.157 (-0.007,0.045)	0.159
2	(ln) MnBP	0.088 (-0.203,0.473)	0.429	-0.144 (-0.156,0.034)	0.205	0.124 (-0.050,0.196)	0.239	0.051 (-0.069,0.108)	0.666	-0.002 (-0.190,0.186)	0.983	-0.054 (-0.135,0.082)	0.629	-0.020 (-0.153,0.129)	0.864	-0.089 (-0.373,0.161)	0.432
	PSS x (ln) MnBP	0.019 (-0.064,0.076)	0.866	0.098 (-0.011,0.028)	0.384	0.062 (-0.018,0.033)	0.556	-0.063 (-0.023,0.013)	0.592	-0.141 (-0.063,0.015)	0.229	-0.072 (-0.030,0.015)	0.519	-0.090 (-0.041,0.018)	0.432	-0.140 (-0.090,0.021)	0.215
1	PSS	-0.049 (-0.039,0.024)	0.639	0.046 (-0.007,0.011)	0.674	0.029 (-0.010,0.013)	0.774	-0.049 (-0.010,0.007)	0.664	-0.024 (-0.020,0.016)	0.833	0.089 (-0.006,0.015)	0.406	0.081 (-0.008,0.018)	0.460	0.126 (-0.010,0.040)	0.245
	(ln) MiBP	0.148 (-0.095,0.567)	0.159	-0.172 (-0.170,0.018)	0.112	0.139 (-0.035,0.206)	0.163	0.049 (-0.068,0.107)	0.661	0.102 (-0.101,0.272)	0.365	-0.068 (-0.143,0.073)	0.523	-0.032 (-0.160,0.120)	0.772	-0.027 (-0.301,0.236)	0.808
	PSS	-0.078 (-0.046,0.022)	0.487	0.033 (-0.008,0.011)	0.771	0.006 (-0.012,0.013)	0.956	-0.056 (-0.011,0.007)	0.641	-0.017 (-0.021,0.018)	0.890	0.129 (-0.005,0.017)	0.259	0.122 (-0.007,0.022)	0.297	0.174 (-0.007,0.048)	0.136
2	(ln) MiBP	0.158 (-0.085,0.589)	0.140	-0.168 (-0.170,0.022)	0.127	0.143 (-0.036,0.211)	0.160	0.054 (-0.068,0.111)	0.640	0.099 (-0.107,0.274)	0.386	-0.085 (-0.153,0.066)	0.431	-0.049 (-0.173,0.110)	0.657	-0.048 (-0.330,0.210)	0.659
	PSS x (ln) MiBP	0.059 (-0.055,0.096)	0.596	0.064 (-0.015,0.028)	0.578	0.045 (-0.022,0.034)	0.675	0.014 (-0.019,0.021)	0.910	-0.037 (-0.049,0.036)	0.756	-0.106 (-0.036,0.013)	0.353	-0.115 (-0.048,0.016)	0.322	-0.132 (-0.096,0.026)	0.253
1	PSS	-0.049 (-0.039,0.024)	0.639	0.046 (-0.007,0.011)	0.674	0.029 (-0.010,0.013)	0.774	-0.049 (-0.010,0.007)	0.664	-0.024 (-0.020,0.016)	0.833	0.089 (-0.006,0.015)	0.406	0.081 (-0.008,0.018)	0.460	0.126 (-0.010,0.040)	0.245
	(ln) OH-MiBP	0.148 (-0.135,0.682)	0.186	-0.134 (-0.185,0.048)	0.245	-0.015 (-0.161, 0.140)	0.888	-0.014 (-0.115,0.102)	0.905	0.024 (-0.208,0.254)	0.842	-0.063 (-0.170,0.096)	0.583	-0.026 (-0.192,0.153)	0.821	-0.119 (-0.500,0.158)	0.303
	PSS	-0.049 (-0.040,0.025)	0.645	0.037 (-0.008,0.011)	0.740	0.022 (-0.011,0.013)	0.834	-0.036 (-0.010,0.007)	0.755	0.002 (-0.018,0.018)	0.983	0.102 (-0.006,0.015)	0.353	0.092 (-0.008,0.019)	0.409	0.145 (-0.009,0.043)	0.187
2	(ln) OH-MiBP	0.148 (-0.141,0.690)	0.192	-0.129 (-0.184,0.053)	0.272	-0.010 (-0.160,0.146)	0.925	-0.023 (-0.120,0.099)	0.848	0.007 (-0.226,0.239)	0.956	-0.070 (-0.176,0.093)	0.543	-0.033 (-0.200,0.150)	0.778	-0.131 (-0.518,0.142)	0.259
	PSS x (ln) OH-MiBP	0.008 (-0.079,0.085)	0.941	0.045 (-0.019,0.028)	0.687	0.041 (-0.024,0.036)	0.688	-0.076 (-0.029,0.014)	0.508	-0.152 (-0.077,0.015)	0.184	-0.077 (-0.036,0.017)	0.480	-0.070 (-0.045,0.024)	0.532	-0.118 (-0.100,0.030)	0.285
1	PSS	-0.049 (-0.039,0.024)	0.639	0.046 (-0.007,0.011)	0.674	0.029 (-0.010,0.013)	0.774	-0.049 (-0.010,0.007)	0.664	-0.024 (-0.020,0.016)	0.833	0.089 (-0.006,0.015)	0.406	0.081 (-0.008,0.018)	0.460	0.126 (-0.010,0.040)	0.245

	(ln) OH-MnBP	0.126 (-0.144,0.568)	0.239	-0.117 (-0.156,0.047)	0.287	0.122 (-0.051,0.209)	0.231	0.050 (-0.073,0.115)	0.659	0.091 (-0.119,0.281)	0.424	-0.075 (-0.156,0.076)	0.492	-0.024 (-0.167,0.133)	0.826	-0.037 (-0.336,0.240)	0.739
	PSS	-0.067 (-0.044,0.023)	0.543	0.036 (-0.008,0.011)	0.753	0.010 (-0.012,0.013)	0.923	-0.055 (-0.011,0.007)	0.641	-0.008 (-0.020,0.018)	0.947	0.116 (-0.005,0.016)	0.306	0.103 (-0.008,0.020)	0.373	0.158 (-0.008,0.046)	0.170
2	(ln) OH-MnBP	0.129 (-0.142,0.579)	0.231	-0.118 (-0.158,0.048)	0.289	0.122 (-0.053,0.211)	0.235	0.053 (-0.073,0.117)	0.646	0.091 (-0.122,0.283)	0.433	-0.081 (-0.160,0.073)	0.458	-0.030 (-0.172,0.131)	0.787	-0.046 (-0.348,0.228)	0.681
	PSS x (ln) OH-MnBP	0.041 (-0.061,0.089)	0.708	0.049 (-0.017,0.026)	0.663	0.044 (-0.022,0.033)	0.673	0.014 (-0.019,0.021)	0.902	-0.066 (-0.054,0.030)	0.575	-0.077 (-0.033,0.016)	0.489	-0.070 (-0.041,0.022)	0.541	-0.097 (-0.086,0.034)	0.393
1	PSS	-0.049 (-0.039,0.024)	0.639	0.046 (-0.007,0.011)	0.674	0.029 (-0.010,0.013)	0.774	-0.049 (-0.010,0.007)	0.664	-0.024 (-0.020,0.016)	0.833	0.089 (-0.006,0.015)	0.406	0.081 (-0.008,0.018)	0.460	0.126 (-0.010,0.040)	0.245
	(ln) OH-MEHP	0.180 (-0.041,0.653)	0.083	-0.150 (-0.170,0.029)	0.162	0.123 (-0.048,0.208)	0.215	-0.030 (-0.105,0.080)	0.790	0.153 (-0.059,0.332)	0.168	-0.110 (-0.173,0.054)	0.301	-0.080 (-0.202,0.093)	0.464	-0.099 (-0.413,0.151)	0.360
	PSS	-0.094 (-0.048,0.019)	0.393	0.043 (-0.008,0.011)	0.705	0.009 (-0.012,0.013)	0.932	-0.055 (-0.011,0.007)	0.647	-0.023 (-0.021,0.017)	0.848	0.121 (-0.005,0.017)	0.286	0.121 (-0.007,0.022)	0.296	0.174 (-0.006,0.047)	0.131
2	(ln) OH-MEHP	0.191 (-0.027,0.677)	0.070	-0.155 (-0.174,0.028)	0.156	0.122 (-0.051,0.210)	0.228	-0.023 (-0.104,0.085)	0.838	0.156 (-0.060,0.338)	0.169	-0.124 (-0.182,0.048)	0.250	-0.094 (-0.214,0.085)	0.393	-0.120 (-0.441,0.126)	0.271
	PSS x (ln) OH-MEHP	0.074 (-0.044,0.090)	0.498	0.068 (-0.014,0.025)	0.549	0.018 (-0.023,0.027)	0.865	0.030 (-0.016,0.020)	0.802	-0.065 (-0.049,0.028)	0.585	-0.058 (-0.028,0.016)	0.606	-0.098 (-0.041,0.016)	0.394	-0.111 (-0.081,0.027)	0.329
1	PSS	-0.049 (-0.039,0.024)	0.639	0.046 (-0.007,0.011)	0.674	0.029 (-0.010,0.013)	0.774	-0.049 (-0.010,0.007)	0.664	-0.024 (-0.020,0.016)	0.833	0.089 (-0.006,0.015)	0.406	0.081 (-0.008,0.018)	0.460	0.126 (-0.010,0.040)	0.245
	(ln) oxo-MEHP	0.116 (-0.165,0.592)	0.265	-0.118 (-0.168,0.048)	0.271	0.167 (-0.018,0.255)	0.089	0.004 (-0.098,0.102)	0.971	0.084 (-0.132,0.294)	0.450	-0.047 (-0.151,0.095)	0.655	0.000 (-0.160,0.159)	0.999	-0.023 (-0.338,0.273)	0.833
	PSS	-0.092 (-0.048,0.020)	0.413	0.045 (-0.008,0.012)	0.696	-0.005 (-0.013,0.012)	0.960	-0.056 (-0.011,0.007)	0.644	-0.018 (-0.021,0.018)	0.880	0.116 (-0.005,0.017)	0.313	0.110 (-0.007,0.021)	0.346	0.166 (-0.008,0.047)	0.154
2	(ln) oxo-MEHP	0.122 (-0.162,0.613)	0.250	-0.134 (-0.179,0.042)	0.223	0.166 (-0.024,0.258)	0.102	0.011 (-0.098,0.108)	0.922	0.095 (-0.127,0.310)	0.406	-0.060 (-0.161,0.091)	0.582	-0.007 (-0.169,0.158)	0.947	-0.037 (-0.363,0.257)	0.734
	PSS x (ln) oxo-MEHP	0.070 (-0.050,0.097)	0.526	0.071 (-0.014,0.028)	0.535	0.021 (-0.024,0.029)	0.838	0.016 (-0.018,0.021)	0.891	-0.067 (-0.053,0.030)	0.575	-0.054 (-0.030,0.018)	0.635	-0.090 (-0.043,0.019)	0.435	-0.106 (-0.086,0.031)	0.355
1	PSS	-0.049 (-0.039,0.024)	0.639	0.046 (-0.007,0.011)	0.674	0.029 (-0.010,0.013)	0.774	-0.049 (-0.010,0.007)	0.664	-0.024 (-0.020,0.016)	0.833	0.089 (-0.006,0.015)	0.406	0.081 (-0.008,0.018)	0.460	0.126 (-0.010,0.040)	0.245
	(ln) cx-MEPP	0.143 (-0.114,0.648)	0.167	-0.114 (-0.168,0.050)	0.287	0.156 (-0.027,0.250)	0.112	0.005 (-0.099,0.103)	0.966	0.090 (-0.126,0.303)	0.415	-0.081 (-0.172,0.076)	0.442	-0.053 (-0.201,0.121)	0.625	-0.069 (-0.408,0.208)	0.520
	PSS	-0.084 (-0.047,0.021)	0.449	0.033 (-0.008,0.011)	0.773	-0.009 (-0.013,0.012)	0.934	-0.069 (-0.012,0.006)	0.561	-0.022 (-0.021,0.017)	0.857	0.109 (-0.006,0.016)	0.339	0.109 (-0.007,0.021)	0.350	0.159 (-0.008,0.046)	0.169
2	(ln) cx-MEPP	0.148 (-0.113,0.667)	0.161	-0.131 (-0.179,0.043)	0.229	0.150 (-0.034,0.249)	0.136	0.006 (-0.101,0.106)	0.960	0.100 (-0.122,0.318)	0.379	-0.093 (-0.182,0.072)	0.389	-0.059 (-0.209,0.120)	0.591	-0.082 (-0.431,0.194)	0.453
	PSS x (ln) cx-MEPP	0.045 (-0.060,0.092)	0.682	0.099 (-0.012,0.031)	0.384	0.053 (-0.021,0.035)	0.613	0.064 (-0.015,0.026)	0.587	-0.052 (-0.053,0.034)	0.662	-0.023 (-0.027,0.022)	0.839	-0.064 (-0.041,0.023)	0.577	-0.068 (-0.080,0.043)	0.549
1	PSS	-0.049 (-0.039,0.024)	0.639	0.046 (-0.007,0.011)	0.674	0.029 (-0.010,0.013)	0.774	-0.049 (-0.010,0.007)	0.664	-0.024 (-0.020,0.016)	0.833	0.089 (-0.006,0.015)	0.406	0.081 (-0.008,0.018)	0.460	0.126 (-0.010,0.040)	0.245
	(ln) OH-MiNP	0.056 (-0.256,0.444)	0.594	-0.172 (-0.179,0.018)	0.106	0.237* (0.030,0.277)	**0.015**	0.052 (-0.070,0.113)	0.640	-0.081 (-0.267,0.124)	0.466	0.063 (-0.079,0.147)	0.551	0.065 (-0.102,0.191)	0.549	0.043 (-0.225,0.337)	0.694
	PSS	-0.047 (-0.040,0.025)	0.657	0.027 (-0.008,0.010)	0.799	0.044 (-0.009,0.014)	0.651	-0.049 (-0.010,0.007)	0.665	-0.030 (-0.020,0.016)	0.789	0.092 (-0.006,0.015)	0.395	0.083 (-0.008,0.019)	0.455	0.129 (-0.011,0.041)	0.243
2	(ln) OH-MiNP	0.053 (-0.265,0.444)	0.616	-0.178 (-0.179,0.013)	0.088	0.242* (0.032,0.281)	**0.014**	0.048 (-0.073,0.113)	0.671	-0.084 (-0.273,0.123)	0.455	0.068 (-0.078,0.150)	0.528	0.068 (-0.101,0.194)	0.534	0.049 (-0.219,0.346)	0.655
	PSS x (ln) OH-MiNP	0.015 (-0.065,0.075)	0.889	0.255* (0.004,0.042)	**0.016**	-0.085 (-0.035,0.014)	0.387	0.069 (-0.013,0.024)	0.544	0.063 (-0.028,0.050)	0.582	0.003 (-0.022,0.023)	0.975	0.035 (-0.024,0.034)	0.754	0.006 (-0.054,0.057)	0.955

1	PSS	-0.049 (-0.039,0.024)	0.639	0.046 (-0.007,0.011)	0.674	0.029 (-0.010,0.013)	0.774	-0.049 (-0.010,0.007)	0.664	-0.024 (-0.020,0.016)	0.833	0.089 (-0.006,0.015)	0.406	0.081 (-0.008,0.018)	0.460	0.126 (-0.010,0.040)	0.245
	(ln) cx-MiNP	-0.025 (-0.397,0.313)	0.814	-0.225* (-0.203,-0.006)	**0.037**	0.177 (-0.013,0.241)	0.077	0.048 (-0.073,0.113)	0.667	0.047 (-0.157,0.240)	0.678	0.030 (-0.099,0.131)	0.783	0.055 (-0.111,0.185)	0.619	0.023 (-0.255,0.314)	0.835
2	PSS	-0.042 (-0.039,0.026)	0.696	-0.024 (-0.010,0.007)	0.810	0.041 (-0.009,0.014)	0.686	-0.081 (-0.012,0.005)	0.475	0.001 (-0.018,0.018)	0.996	0.080 (-0.007,0.014)	0.466	0.073 (-0.009,0.018)	0.514	0.126 (-0.011,0.041)	0.261
	(ln) cx-MiNP	-0.034 (-0.422,0.309)	0.759	-0.156 (-0.168,0.022)	0.131	0.168 (-0.022,0.240)	0.102	0.082 (-0.060,0.129)	0.474	0.022 (-0.184,0.223)	0.847	0.041 (-0.096,0.140)	0.713	0.065 (-0.109,0.196)	0.568	0.026 (-0.258,0.326)	0.817
	PSS x (ln) cx-MiNP	-0.039 (-0.086,0.059)	0.720	0.349* (0.013,0.051)	**0.001**	-0.048 (-0.032,0.020)	0.640	0.176 (-0.004,0.033)	0.128	-0.124 (-0.062,0.019)	0.290	0.048 (-0.018,0.029)	0.663	0.044 (-0.024,0.036)	0.700	0.005 (-0.057,0.059)	0.966
1	PSS	-0.049 (-0.039,0.024)	0.639	0.046 (-0.007,0.011)	0.674	0.029 (-0.010,0.013)	0.774	-0.049 (-0.010,0.007)	0.664	-0.024 (-0.020,0.016)	0.833	0.089 (-0.006,0.015)	0.406	0.081 (-0.008,0.018)	0.460	0.126 (-0.010,0.040)	0.245
	(ln) ΣDiBP	0.143 (-0.047,0.249)	0.179	-0.160 (-0.073,0.011)	0.144	0.142 (-0.016,0.093)	0.162	0.060 (-0.029,0.050)	0.599	0.105 (-0.045,0.123)	0.358	-0.074 (-0.065,0.031)	0.492	-0.029 (-0.071,0.054)	0.793	-0.030 (-0.136,0.103)	0.783
2	PSS	-0.073 (-0.045,0.023)	0.515	0.031 (-0.008,0.011)	0.787	0.006 (-0.012,0.013)	0.951	-0.055 (-0.011,0.007)	0.645	-0.011 (-0.020,0.018)	0.927	0.123 (-0.005,0.017)	0.280	0.114 (-0.007,0.021)	0.327	0.167 (-0.007,0.047)	0.151
	(ln) ΣDiBP	0.147 (-0.046,0.254)	0.171	-0.154 (-0.073,0.012)	0.162	0.145 (-0.015,0.094)	0.155	0.061 (-0.029,0.050)	0.598	0.100 (-0.047,0.122)	0.383	-0.082 (-0.067,0.030)	0.452	-0.037 (-0.073,0.052)	0.740	-0.040 (-0.142,0.098)	0.719
	PSS x (ln) ΣDiBP	0.048 (-0.026,0.040)	0.668	0.069 (-0.006,0.012)	0.543	0.045 (-0.009,0.015)	0.671	0.011 (-0.008,0.009)	0.929	-0.055 (-0.023,0.014)	0.644	-0.092 (-0.015,0.006)	0.415	-0.097 (-0.020,0.008)	0.401	-0.117 (-0.040,0.013)	0.307
1	PSS	-0.049 (-0.039,0.024)	0.639	0.046 (-0.007,0.011)	0.674	0.029 (-0.010,0.013)	0.774	-0.049 (-0.010,0.007)	0.664	-0.024 (-0.020,0.016)	0.833	0.089 (-0.006,0.015)	0.406	0.081 (-0.008,0.018)	0.460	0.126 (-0.010,0.040)	0.245
	(ln) ΣDnBP	0.097 (-0.082,0.216)	0.372	-0.140 (-0.069,0.016)	0.211	0.125 (-0.021,0.087)	0.228	0.064 (-0.028,0.050)	0.583	0.036 (-0.071,0.097)	0.755	-0.048 (-0.059,0.038)	0.662	-0.003 (-0.063,0.062)	0.981	-0.055 (-0.148,0.090)	0.625
2	PSS	-0.056 (-0.042,0.024)	0.603	0.029 (-0.008,0.011)	0.794	0.015 (-0.011,0.013)	0.883	-0.039 (-0.010,0.007)	0.735	0.001 (-0.018,0.019)	0.990	0.107 (-0.005,0.016)	0.335	0.099 (-0.008,0.020)	0.380	0.155 (-0.008,0.044)	0.166
	(ln) ΣDnBP	0.099 (-0.082,0.219)	0.366	-0.134 (-0.068,0.017)	0.236	0.130 (-0.020,0.089)	0.215	0.060 (-0.029,0.050)	0.608	0.027 (-0.074,0.094)	0.817	-0.055 (-0.060,0.037)	0.625	-0.009 (-0.066,0.060)	0.934	-0.065 (-0.154,0.085)	0.564
	PSS x (ln) ΣDnBP	0.027 (-0.027,0.034)	0.800	0.084 (-0.005,0.012)	0.449	0.056 (-0.008,0.014)	0.583	-0.047 (-0.010,0.006)	0.684	-0.118 (-0.026,0.008)	0.308	-0.079 (-0.013,0.006)	0.475	-0.085 (-0.018,0.008)	0.448	-0.129 (-0.038,0.010)	0.247
1	PSS	-0.049 (-0.039,0.024)	0.639	0.046 (-0.007,0.011)	0.674	0.029 (-0.010,0.013)	0.774	-0.049 (-0.010,0.007)	0.664	-0.024 (-0.020,0.016)	0.833	0.089 (-0.006,0.015)	0.406	0.081 (-0.008,0.018)	0.460	0.126 (-0.010,0.040)	0.245
	(ln) ΣDEHP	0.163 (-0.037,0.307)	0.122	-0.123 (-0.078,0.021)	0.258	0.143 (-0.018,0.108)	0.155	-0.003 (-0.046,0.045)	0.976	0.110 (-0.050,0.145)	0.332	-0.098 (-0.082,0.030)	0.362	-0.063 (-0.094,0.052)	0.569	-0.086 (-0.194,0.083)	0.431
2	PSS	-0.093 (-0.048,0.019)	0.401	0.046 (-0.008,0.012)	0.686	-0.001 (-0.012,0.012)	0.994	-0.056 (-0.011,0.007)	0.636	-0.024 (-0.021,0.017)	0.842	0.117 (-0.005,0.017)	0.304	0.116 (-0.007,0.021)	0.316	0.168 (-0.007,0.047)	0.146
	(ln) ΣDEHP	0.163 (-0.038,0.308)	0.123	-0.123 (-0.078,0.021)	0.260	0.143 (-0.018,0.109)	0.157	-0.003 (-0.047,0.045)	0.977	0.110 (-0.050,0.145)	0.335	-0.098 (-0.082,0.030)	0.365	-0.063 (-0.094,0.052)	0.569	-0.086 (-0.194,0.084)	0.431
	PSS x (ln) ΣDEHP	0.064 (-0.022,0.041)	0.556	0.065 (-0.007,0.012)	0.563	0.026 (-0.010,0.013)	0.806	0.029 (-0.007,0.010)	0.802	-0.061 (-0.023,0.013)	0.604	-0.044 (-0.012,0.008)	0.693	0.090 (-0.008,0.019)	0.413	-0.098 (-0.037,0.014)	0.385
1	PSS	-0.049 (-0.039,0.024)	0.639	0.046 (-0.007,0.011)	0.674	0.029 (-0.010,0.013)	0.774	-0.049 (-0.010,0.007)	0.664	-0.024 (-0.020,0.016)	0.833	0.089 (-0.006,0.015)	0.406	0.081 (-0.008,0.018)	0.460	0.126 (-0.010,0.040)	0.245
	(ln) ΣDiNP	0.123 (-0.098,0.323)	0.290	-0.228* (-0.121,0.001)	**0.054**	0.118 (-0.033,0.115)	0.273	-0.029 (-0.057,0.045)	0.815	0.105 (-0.164,0.065)	0.392	-0.064 (-0.084,0.048)	0.591	-0.026 (-0.099,0.079)	0.829	-0.042 (-0.202,0.141)	0.727
2	PSS	-0.026 (-0.039,0.031)	0.825	0.031 (-0.008,0.011)	0.777	0.038 (-0.010,0.014)	0.727	-0.038 (-0.010,0.007)	0.764	-0.106 (-0.027,0.011)	0.394	0.099 (-0.006,0.015)	0.416	0.093 (-0.009,0.020)	0.452	0.121 (-0.014,0.043)	0.322

	(ln) ΣDiNP	0.119 (-0.106,0.325)	0.314	-0.217* (-0.115,0.000)	**0.051**	0.120 (-0.033,0.117)	0.271	-0.032 (-0.058,0.045)	0.801	-0.115 (-0.171,0.062)	0.353	-0.053 (-0.082,0.052)	0.659	-0.015 (-0.096,0.085)	0.901	-0.028 (-0.194,0.154)	0.818
	PSS x (ln) ΣDiNP	-0.015 (-0.043,0.038)	0.901	0.373 (0.008,0.029)	**0.001**	-0.118 (-0.022,0.006)	0.278	0.078 (-0.007,0.013)	0.534	0.118 (-0.011,0.032)	0.342	-0.054 (-0.015,0.010)	0.655	-0.013 (-0.018,0.016)	0.914	-0.029 (-0.036,0.029)	0.809
1	PSS	-0.049 (-0.039,0.024)	0.639	0.046 (-0.007,0.011)	0.674	0.029 (-0.010,0.013)	0.774	-0.049 (-0.010,0.007)	0.664	-0.024 (-0.020,0.016)	0.833	0.089 (-0.006,0.015)	0.406	0.081 (-0.008,0.018)	0.460	0.126 (-0.010,0.040)	0.245
	(ln) ΣLMWP	0.021 (-0.138,0.168)	0.846	-0.121 (-0.067,0.020)	0.283	0.076 (-0.035,0.076)	0.468	0.142 (-0.015,0.064)	0.224	0.057 (-0.065,0.107)	0.625	-0.037 (-0.058,0.041)	0.739	0.058 (-0.048,0.080)	0.612	-0.025 (-0.136,0.110)	0.830
	PSS	-0.056 (-0.041,0.024)	0.602	0.033 (-0.008,0.010)	0.757	0.024 (-0.010,0.013)	0.812	-0.050 (-0.010,0.007)	0.658	-0.013 (-0.019,0.017)	0.910	0.100 (-0.006,0.015)	0.356	0.088 (-0.008,0.019)	0.424	0.143 (-0.009,0.042)	0.192
2	(ln) ΣLMWP	0.026 (-0.137,0.173)	0.818	-0.116 (-0.066,0.021)	0.305	0.076 (-0.036,0.077)	0.474	0.142 (-0.016,0.065)	0.228	0.053 (-0.067,0.106)	0.657	-0.045 (-0.060,0.040)	0.692	0.051 (-0.050,0.079)	0.656	-0.036 (-0.142,0.103)	0.754
	PSS x (ln) ΣLMWP	0.055 (-0.024,0.042)	0.602	0.150 (-0.003,0.016)	0.163	0.022 (-0.011,0.013)	0.828	-0.029 (-0.010,0.007)	0.797	-0.121 (-0.028,0.008)	0.282	-0.094 (-0.015,0.006)	0.381	-0.090 (-0.019,0.008)	0.410	-0.147 (-0.044,0.008)	0.173
1	PSS	-0.049 (-0.039,0.024)	0.639	0.046 (-0.007,0.011)	0.674	0.029 (-0.010,0.013)	0.774	-0.049 (-0.010,0.007)	0.664	-0.024 (-0.020,0.016)	0.833	0.089 (-0.006,0.015)	0.406	0.081 (-0.008,0.018)	0.460	0.126 (-0.010,0.040)	0.245
	(ln) ΣHMWP	0.111 (-0.076,0.256)	0.288	-0.199 (-0.090,0.002)	0.063	0.222* (0.009,0.126)	**0.025**	0.019 (-0.040,0.047)	0.865	0.038 (-0.077,0.108)	0.737	-0.009 (-0.056,0.051)	0.933	0.020 (-0.063,0.075)	0.858	-0.013 (-0.141,0.125)	0.905
	PSS	-0.068 (-0.044,0.023)	0.539	-0.003 (-0.009,0.009)	0.976	0.032 (-0.010,0.014)	0.756	-0.066 (-0.011,0.006)	0.573	-0.016 (-0.020,0.018)	0.895	0.102 (-0.006,0.016)	0.366	0.097 (-0.008,0.020)	0.397	0.150 (-0.009,0.044)	0.187
2	(ln) ΣHMWP	0.112 (-0.078,0.255)	0.293	-0.212* (-0.092,-0.001)	**0.046**	0.223* (0.008,0.127)	**0.026**	0.018 (-0.040,0.047)	0.870	0.041 (-0.077,0.111)	0.721	-0.011 (-0.057,0.051)	0.919	0.019 (-0.064,0.076)	0.864	-0.015 (-0.142,0.124)	0.892
	PSS x (ln) ΣHMWP	0.048 (-0.026,0.041)	0.658	0.216* (0.000,0.018)	**0.049**	-0.048 (-0.015,0.009)	0.635	0.062 (-0.006,0.011)	0.596	-0.037 (-0.022,0.016)	0.752	-0.045 (-0.013,0.009)	0.686	-0.064 (-0.018,0.010)	0.576	-0.086 (-0.037,0.016)	0.448

Note: all models adjusted for confounders: gestational week, age, BMI, active and passive smoking

Legend: 1-main effects, 2-inetraction effects, β-standardized coefficient, CI-confidence interval, cx-MEPP-mono(2-ethyl-5-carboxypentyl) phthalate, cx-MiNP-mono(carboxy-methyl-heptyl) phthalate, FSH-follicle-stimulating hormone, FT3-free triiodothyronine, FT4-free thyroxine, LH-luteinizing hormone, ln - log-transformed values, MEP-monoethyl phthalate, MiBP-mono-iso-butyl phthalate, MnBP- mono-n-butyl phthalate, OH-MEHP-mono(2-ethyl-5-hydroxyhexyl) phthalate, OH-MiBP-mono(hydroxy-iso-butyl) phthalate, OH-MnBP- mono(hydroxy-n-butyl) phthalate, OH-MiNP-mono(hydroxyl-methyl-octyl) phthalate, oxo-MEHP-mono(2-ethyl-5-oxohexyl) phthalate, p-value of statistical significance, PSS-perceived stress scale, TSH-thyroid-stimulating hormone, ΣDiBP-molar sum of di-iso-butyl phthalate metabolites (MiBP+OH-MiBP), ΣDnBP-molar sum of di-n-butyl phthalate metabolites (MnBP+OH-MnBP), ΣDEHP-molar sum of di(2-ethylhexyl) phthalate metabolites (OH-MEHP+oxo-MEHP+cx-MEPP), ΣDiNP-molar sum of di-iso-nonyl phthalate metabolites (OH-MiNP+cx-MiNP), ΣLMWP-molar sum of low molecular-weight phthalate metabolites (MEP+MiBP+MnBP+OH-MiBP+OH-MnBP), ΣHMWP-molar sum of high molecular-weight phthalate metabolites (OH-MEHP+oxo-MEHP+cx-MEPP+OH-MiNP+cx-MiNP).

* significant association (*p*≤0.05)
